# Novel methods of qualitative analysis for health policy research

**DOI:** 10.1186/s12961-018-0404-z

**Published:** 2019-01-14

**Authors:** Mireya Martínez-García, Maite Vallejo, Enrique Hernández-Lemus, Jorge Alberto Álvarez-Díaz

**Affiliations:** 1 Sociomedical Research Department, National Institute of Cardiology, Juan Badiano 1, Mexico City, 14080 Mexico; 2 Computational Genomics Division, National Institute of Genomic Medicine, Periférico Sur 4809, Mexico City, 14610 Mexico; 3 Medical School, Autonomous Metropolitan University, Calzada del Hueso 1100, Mexico City, 04960 Mexico

**Keywords:** Data mining, Algorithms, Complex networks, Healthcare economics and organisations, Social control policies, Health policy

## Abstract

**Background:**

Currently, thanks to the growing number of public database resources, most evidence on planning and management, healthcare institutions, policies and practices is becoming available to everyone. However, one of the limitations for the advancement of data and literature-driven research has been the lack of flexibility of the methodological resources used in qualitative research. There is a need to incorporate friendly, cheaper and faster tools for the systematic, unbiased analysis of large data corpora, in particular regarding the qualitative aspects of the information (often overlooked).

**Methods:**

This article proposes a series of novel techniques, exemplified by the case of the role of Institutional Committees of Bioethics to (1) massively identify the documents relevant to a given issue, (2) extract the fundamental content, focusing on qualitative analysis, (3) synthesize the findings in the published literature, (4) categorize and visualize the evidence, and (5) analyse and report the results.

**Results:**

A critical study of the institutional role of public health policies and practices in Institutional Committees of Bioethics was used as an example application of the method. Interactive strategies were helpful to define and conceptualise variables, propose research questions and refine research interpretation. These methods are additional aids to systematic reviews, pre-coding schemes and construction of a priori diagrams to survey and analyse social science literature.

**Conclusions:**

These novel methods have proven to facilitate the formulation and testing of hypotheses on the subjects to be studied. Such tools may allow important advances going from descriptive approaches to decision-making and even institutional assessment and policy redesign, by pragmatic reason of time and costs.

**Electronic supplementary material:**

The online version of this article (10.1186/s12961-018-0404-z) contains supplementary material, which is available to authorized users.

## Background

### Complexities of policy analysis

Healthcare institutions are complex organisations whose procedures, activities and, ultimately, outcomes should be assessed constantly in order to optimise their functionality in ever-changing environments [1]. There are several ways to perform qualitative analysis of Health Care Institutions Policies and Practices (HCIPP), including ethnography, ethnomethodology, phenomenology, action research, grounded theory, critical discourse analysis, and evidence-based science, among others [2]. The Qualitative Research Methodology (QRM), for instance, uses data collected to discover or refine research questions because, usually, performance variables are not fully conceptualised or completely defined [3, 4].

As with all research strategies, choosing the best QRM is vital to obtaining the desired results in HCIPP analysis. Computerized Qualitative Analysis of Discourse (CQAD), for instance, is used to extract and synthesise descriptions of search, selection, quality appraisal, analysis and synthesis methods. Additionally, evidence-based non-systematic literature reviews (NSLR), rapid reviews, scoping studies and research syntheses have gained wide acceptance in the QRM [5].

CQAD and NSLR have some degree of empirical support and classifying evidence of their epistemological strength; both converge in the analytic phase, sharing methodologies for decontextualising and recontextualising data, coding, sorting, identifying themes and relationships, and drawing conclusions [2]. At this stage, it is useful to assess the strengths and limitations of current approaches to policy analysis and to address how improvement can be achieved in this regard.

### Advantages and disadvantages of traditional approaches

A literature search is a key step in carrying out a good reliable HCIPP research. It helps in formulating or refining a research question and planning the strategies of study [6]. Access to the most relevant articles, with maximum evidence, in a shorter time and with less cost is essential for HCIPP analysis.

Bioethics literature is vast. Researchers use CQAD and NSLR to examine patterns in documents in a replicable and systematic manner. On the one hand, CQAD is used to automate the classification and coding categories in texts or in preparing sets of texts for building up inferences. Additionally, lesser time and cost are advantages, in contrast to the manual limitative analysis of just measuring the number of words and lines. The larger disadvantage of CQAD is the dependence on subjective impressions of a reader [7, 8].

On the other hand, two types of non-systematic review have been discussed in relation to bioethics literature, namely Introductory Reviews of Bioethics Literature and Critical Interpretive Reviews of Bioethics literature. These approaches have been quite popular recently since they are faster and easier to implement than systematic reviews of the literature [9, 10]. However, some of them have brought scientific and methodological controversies about transparency, rigor, comprehensiveness and reproducibility. Further, these approaches have disadvantages related to the insufficiently focused review scope, diversity of terminology in order to identify all relevant publications and quality assessment [5, 11].

### Introducing novel methods

This article on novel methods of qualitative analysis is aimed towards policy-makers, bioethics health professionals and researchers. The model has been proposed by pragmatic reasons of time and costs. Many of the processes underlying institutional policies and practices have not been properly investigated; thus, there is a need to incorporate QRM frameworks for such research. Consequently, to address the phenomenon of institutional analysis and to account for its relationship to public health, a systematic model of critical analysis is proposed and exemplified by the case of the role of Institutional Committees of Bioethics (ICB).

## Methods

### Aims of this work and case study outline

As a case study to introduce our methodological proposal, we will analyse the case of HCIPP of **ICB**. In this subsection, we will provide some information regarding the choice of this case study and the foundations for its analysis.

The bioethical discourse in public policy establishes an important part of the practice in the public healthcare institutions as is the case of ICB. This is so, since recently, science and technology have been increasingly reassessed in ethical terms [12–14]. Ethics has become the decisive semantic form in which government discourses are carried out with greater political relevance, and has since become the dominant discourse [15]. As a branch of applied ethics, Bioethics has become the political medium for the creation of a moral economy where value commitments are made capable of legitimising the regulatory policies necessary to maintain public confidence in biomedical science and healthcare [16].

A growing number of studies have explored the role of ICB in various fields, from academic and biotechnological, to medical and legal [13, 14]. The strategies for the study of the literary forms of governance of ICB have been separated by opinions, reports, guidelines and consensus statements, focused more on who does things, how and why they do them, yet in isolated form, even when CQAD and NSLR have been implemented [17]. With this scenario in mind, laying out a comprehensive method has been necessary to improve ICB policy and practice analyses.

### A three-stage research design

**Fig. 1:**
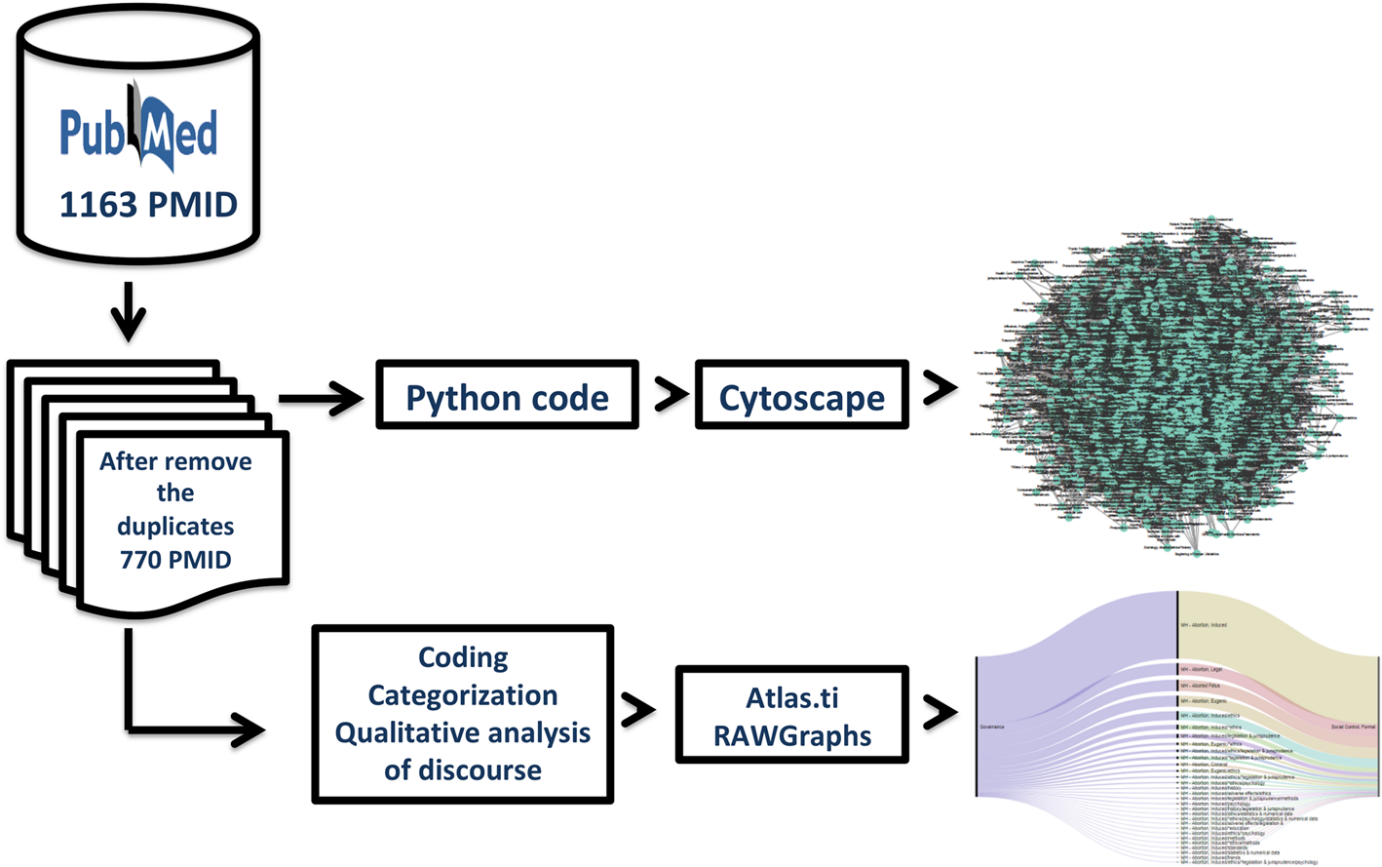
Flow diagram showing the steps followed in this work

To address the methodological approach already sketched, we will proceed along the following lines. First, the construction of a preliminary corpus with texts extracted from the Medical Literature Analysis and Retrieval System Online (MEDLINE) PubMed database (Stage I). At this stage, no biased decision as to the content of the corpus was made, except, of course, on the pertinence to the problem under study. Second, two textual exploration techniques were performed simultaneously, namely tracing of the corpus of Medical Subject Headings (MeSH) terms for the construction of a semantic network (Stage II) and an inspection of the corpus MEDLINE to identify a priori codes and categories by means of both manual and automated by CQAD (Stage III). Finally, the main findings were discussed in order to contribute to the generation of a systematic and unbiased methodological proposal to address this social phenomenon. The proposed research strategy is set out below (as it can be seen in Fig. 1).

First, build a preliminary corpus with the documents extracted from the MEDLINE PubMed database. Second, two text exploration processes were carried out in parallel, namely (1) an exploration of the corpus of MeSH terms for the construction of a semantic network and (2) an exploration of the content obtained from the PubMed corpus to identify preliminary codes and categories. Both by manual and automated processes (cytoscape and Atlas.ti softwares) were used in both cases. Third, two types of visualisations were obtained from the previous processes (semantic network and alluvial diagrams).

#### Stage I: Documental corpus identification

With the rapid expansion of scientific research, an effective search and the massive integration of new knowledge have become difficult. The development of methods and tools available to researchers has been one of the main lines of research in computer science. Some massive document search tools have been made more precise and a large part of the integrated graphic visualisation tools to show the relationships between authors, topics and appointments, among others, are now available. These innovative search and mass visualisation systems not only facilitate the systematisation of information, they can also help the social sciences researcher to develop a conceptual mapping to identify categories of analysis, as well as emerging categories. With this approach, the conceptualisation of the research problem can be improved, as well as enriching the abstractions and representations of the phenomena in question.

Given that the researchers in social sciences have a lot to read, it is essential not to spend much time searching for potential information. Therefore, it is recommended that these systems of massive document retrieval may be increasingly used, which at the same time allows a simple and rapid systematisation, categorisation and codification of the required knowledge. Several tools, such as the mapping technique, have been developed to graphically represent relationships between document knowledge through networks of concepts, also called semantic networks. These networks consist of nodes and links, wherein nodes represent concepts and links represent connections between documents [18–20].

In this work, a MEDLINE (PubMed) search was first performed to massively identify the documents that may contain terms related to the case study used as an example for the development of this methodology. The search terms entered in these engines were:

- Institutionalization of bioethics and public health policy
- Institutionalization of bioethics and public health
- Institutionalization of bioethics
- Bioethics committees and public health policy
- Bioethics committees and public policy

#### Stage II: Semantic network curation

Once this corpus of information was obtained, the two referred processes of exploration of the texts were performed. Firstly, the use of ontology retrieved from the MeSH terms. The importance of this exploration process lies in the compartmentalisation of information, a property that allows the implementation of algorithmic approaches for its analysis, an extremely valuable resource for the massive mining of literature such as the one implemented in this work.

This stage consisted in the qualitative analysis of data extracted from PubMed MEDLINE. In recent times, network-based approaches (semantic or ontology-based networks) to understand complex social, political, biological and technological issues have been developed [21]. Such approaches are useful since they allow the researcher to have an unbiased, integrated view to discover associations and interactions between the relevant instances involved.

Connectivity maps are built so that source and target nodes are the core concepts in a given corpus and links between these correspond to co-existence of the concepts on a given database, the more instances of repeat co-occurrence, the stronger the link and hence the closer the connection between these concepts (given the underlying corpus, of course).

A previously validated Python code was used to design a reference structure and make way for network analysis with Cytoscape, an open source software platform, to analyse and visualise complex networks of interactions, in this case, semantic. All the source code for general text processing can be found at https://github.com/CSB-IG/literature/tree/master/text_processing. The calculation of the underlying literature-based measures can be found at https://github.com/CSB-IG/bibliometrics.

Once the structured file was processed in a network with the NetworkX Python library, some connectivity maps were built, so that the nodes represented the MeSH terms, and the links between these were the documents (PMID of each publication) that shared MeSH terms among them.

#### Stage III: Content exploration

The second analysis was performed through the manual selection text content technique, an approach to computer aided CQAD, implemented by the Atlas.ti software. CQAD is a systematic coding and categorisation approach used to explore large amounts of textual and discourse information to determine trends and patterns of words used, their frequency, relationships and structures [22–24]. Atlas.ti is a computer programme for the analysis of qualitative data that allows the import and encoding of textual data as a category that designates broadly semiotic elements [25–27].

All the data within the Atlas.ti tool were organised into a hermeneutic unit (a repository of pdf documents) and, from it, citations, codes and groups of codes were built. The citations were the places where the ideas were stored (a physical location) and the codes were the spaces to store the categories (a way of labelling certain aspects of the data and classifying the information). The Atlas.ti query functions were used to search for coding patterns in the project database.

We used a combined deductive and inductive strategy to construct codes and categories; the deductive approach as a priori defined categories based on a theory or framework and inductive approach as a posteriori built codes and categories [8].

The operationalisation includes the materialisation of the speeches. In the case of the example, the HCIPP analysis and its relation to the sociopolitical speeches of the ICB, the variables can materialise from an a priori category in the bio-power instruments such as a patronage, clientelism, simulation or authoritarianism. The next step was to adapt these conceptual categories to compare them with the corpus of texts and discourses; while this empirical process was being carried out, other pertinent categories emerged that completed the analysis.

Once the information sources of the database were selected in the Atlas.ti hermeneutic unit, an initial scan of collected information was carried out and the codes and categories were constructed, for the evidentiary stage, for which it was called the Protocol of codification (a table of codes and categories systematised by the software Atlas.ti). The code table and a category map were generated, which was visualised by means alluvial diagram (RAW Graphs) [28].

These strategies were based on the notion of discovering a possible covert bio-political structure behind an institutionalised symbolic order [29]. The symbolic struggles and power can determine these hidden orders that exist in all social reality, inherent in different fields of knowledge and capable of revealing the role (or roles) of a certain organisation but veiled by a system of politically correct discourses and endorsed by the scientific bodies in the corpus of related literature.

## Results

### Stage I Results: documental corpus identification

The search terms entered in the search engine were (the search was made on January 3, 2018):

- Institutionalization of bioethics and public health policy (4 results)
- Institutionalization of bioethics and public health (18 results)
- Institutionalization of bioethics (42 results)
- Bioethics committees and public health policy (433 results)
- Bioethics committees and public policy (666 results)

The documents were retrieved in plain text (txt) format, a corpus with 770 records was formed, after removing the duplicates (*n*= 393), each one of these constituted, among other elements, an identifier of the MEDLINE (PubMed) database (PMID), title, summary, date of publication, name and place of ascription of the authors, as well as the country where the research work took place.

### Stage II Results: semantic network analysis

To understand the structure of the network and the interrelationships between its elements, a brief analysis of the connectivity patterns was carried out, such as the number of nodes and the number of connections.

**Fig. 2:**
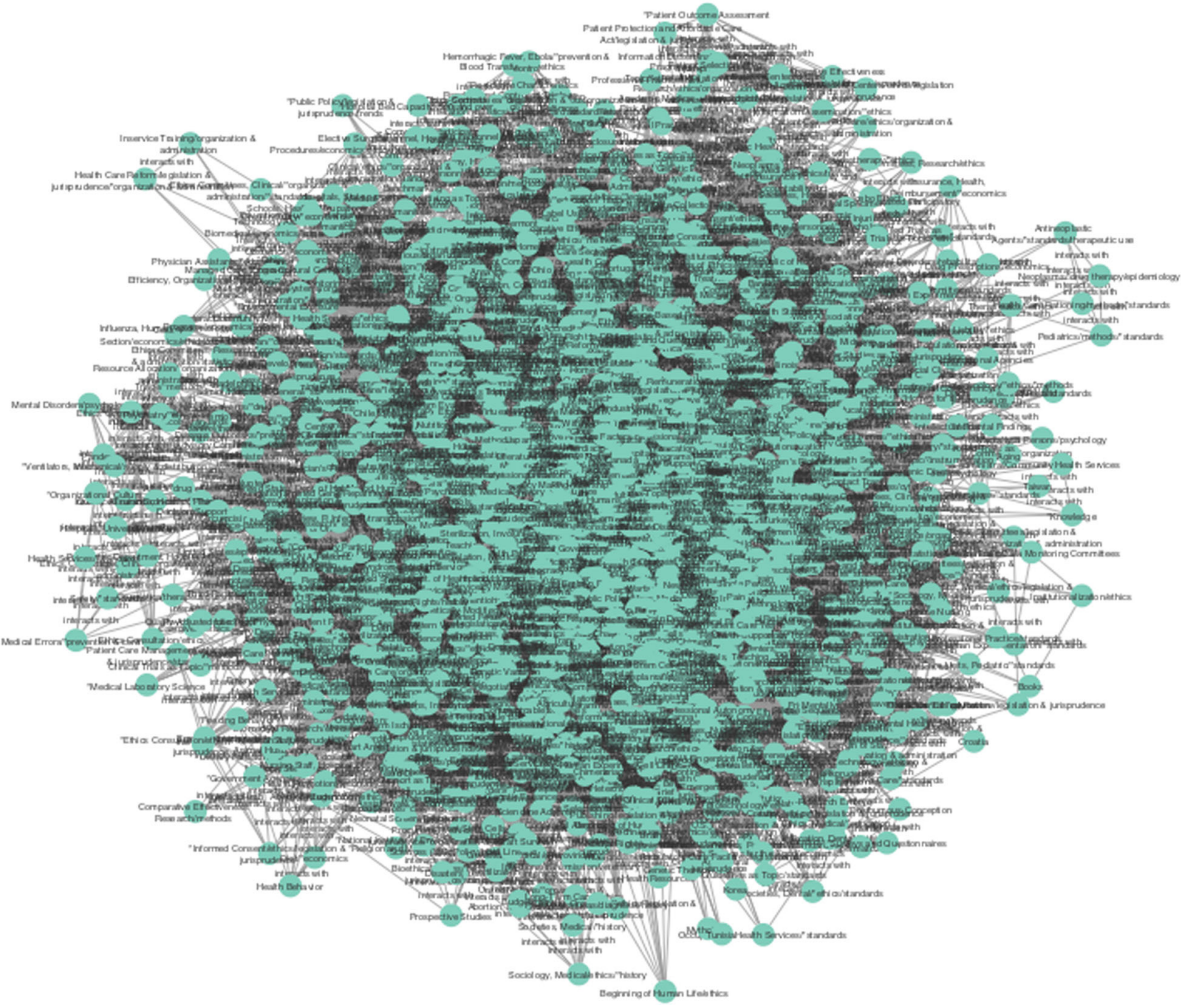
In this Global network a quite complex graph is visually appreciated, composed of a single connected element, with a large number of nodes (MeSH terms) (1996) as well as a large number of links (63,488)

Figure 2 shows a complex network, composed of a single connected element, with a large number of nodes, there is a relatively large number (1996) of interrelated concepts connected by a vast amount (63,488) of interconnections, responsible for the overall conceptual richness of the underlying discourse. Being this a highly clustered graph (average clustering coefficient is 0.808, meaning that more than 80% of all possible triplets of related concepts are actually present in the network), most of the concepts behind the published literature on ICB are tightly connected.

Moreover, the network centralisation statistic equals 0.917, which is indicative that a relatively small number of concepts are responsible for the highly connected structure of the network. If we look at the actual data behind Fig. 2 (To look up for network topological structure and node/link statistics, please refer to Additional file 1), we can identify concepts such as Human, United States, Advisory Committees, and Public Policy (with two instances) that possess more than a thousand direct conceptual associations to other terms in this context.

Humans with 1891 connections results obvious since ethics is a human construct, the case of the term United States (1288 links) reflects the fact that an important corpus of research has been published by United States-based researchers and –more importantly– within the geopolitical context of American public health and policy. This fact has to be taken seriously into account when analysing public policy under different national contexts by realising that the public research corpora will be heavily biased towards the United States-like schemes.

The fact that Advisory Committees (1077 links) and Public Policy (with 907 links when considered as a central concept and 792 links as a secondary category) show so many links is also not surprising. What may result more surprising is the fact that important social and philosophical concepts such as Institutionalization/ethics, Communities/health services and Ethics, Medical/education are at the low end of the distribution, all of them with at most 5 links.

If we consider that, on average, each concept in this semantic network is associated to 63.315 other concepts (in the world corpora of published literature in the field as represented in this analysis), the important issues of institutionalisation of ethics, community outreach and medical education are severely disconnected from the main discussions in the current literature.

**Fig. 3:**
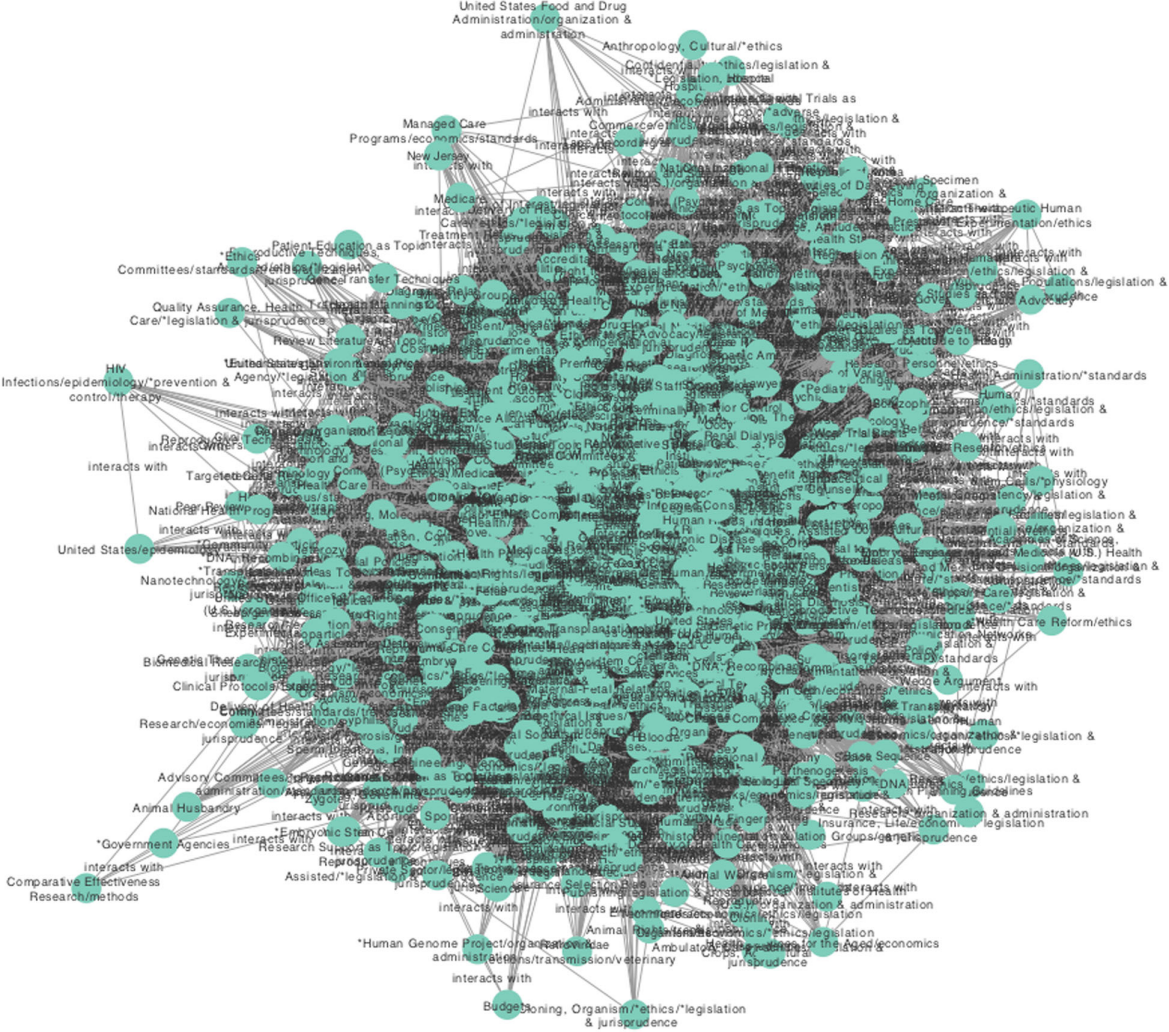
This figure shows the subnet based on the term MeSH Government Regulation and its first neighbours

**Fig. 4:**
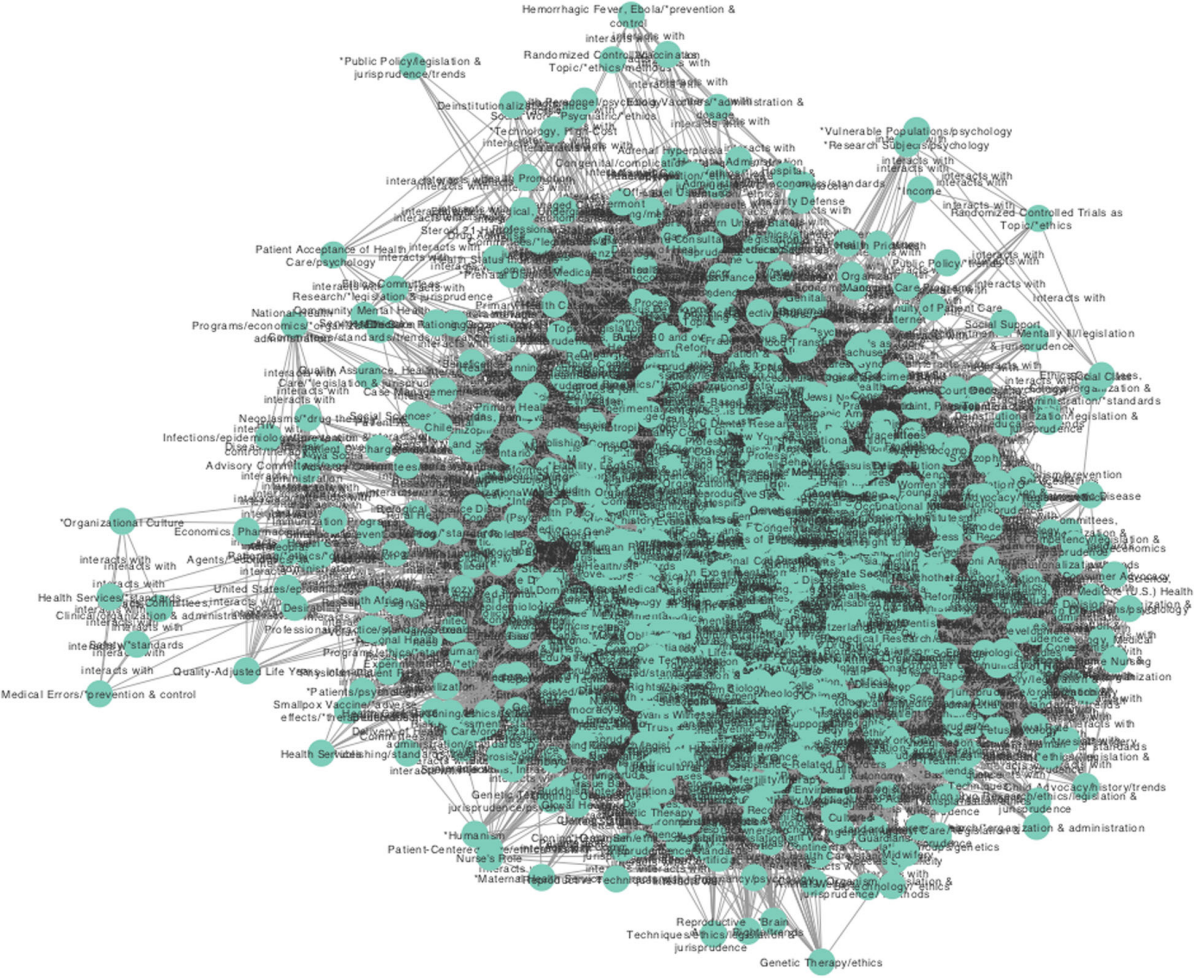
This figure shows the subnet based on the term MeSH Social Control, Formal and its first neighbours

As the main objective of our work is to show the example of ICB and give an account of their relationship with public policies, we analysed the entire network in a context identified by a priori MeSH terms with the largest number of connections. In this way, we decided to build two subnets based on the following MeSH terms and their first neighbours: Government Regulation (GR-683 connections), Social Control and Formal (SCF-451 connections) see Figs. 3 and 4. This is so, since, aside from the already commented –and somehow obvious– cases of Humans, United States, Advisory Committees and Public Policy terms, Government Regulation and Social Control are highly connected concepts central to understanding the role played by the ICB.

Moving on to discuss the Government Regulation sub-network, it is also a large (952 concepts/nodes and 44,091 associations/links) and quite clustered network (clustering coefficient equal to 0.741), with a high centralisation (0.883), which indicates that there are really important concepts (hubs) that link together with most other concepts.

Among such highly central terms, we can mention, aside from the already mentioned global hubs, Federal Government with 587 links, Bioethics (i.e. Bioethics as a secondary subject) with 478 links, as well as Advisory Committees (453 connections), Risk Assessment (441 connections) and Informed Consent (439 links). Interestingly, concepts such as *Government Agencies, Policy and Budget are all under-represented concepts with 10 or less connections in this sub-network, as compared with the average number of neighbours, which is 92.628.

In relation to the Social Control, Formal sub-network, it consists of 1133 concepts and 51,142 relations. With a clustering coefficient of 0.741, and a centralisation of 0.903, this network presented similar connectivity features as the already discussed networks, namely a densely interconnected graph with a small number of highly central concepts. In this case, emerging concepts are Social Values with 567 connections, Government Regulation with 555, and *Bioethical issues with 507 connections, whereas interesting under-represented terms are Health Services/*standards, Safety/*standards and Organisational Culture with 6 links each (versus links on average for this network).

The above described analysis represents just a glimpse of the vast amount of contextual information that can be derived from semantic network studies and will serve as a systematic method for educated hypothesis generations. Such hypotheses can be further pursued by following the tenets of social analysis of discourse and policy assessment, as well as other methods of analysis in the social sciences.

### Stage III Results: content analysis codification protocol

**Fig. 5:**
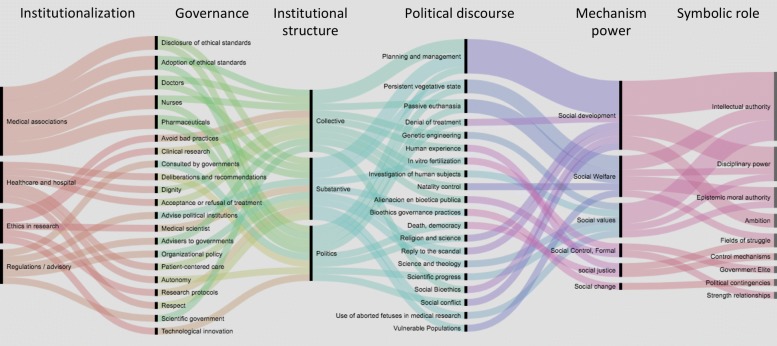
Model for dimensions of analysis. Columns: (1) Forms of institutionalisation, (2) Forms of governance, (3) Institutional structure, (4) Political discourse, (5) Mechanism power and (6) Symbolic role

One way to know the symbolic order was to establish the dimensions of analysis by categories and codes, see the first column of Fig. 5. These were determined in at least six possible categories and each of them with multiple codes, which would correspond to the role of the ICB as an organisational model of a public policy.

The categories were reconstructed from the information collected and a priori analysed, using a content analysis technique according to the recommended procedures by Fairclough [30]. This analysis, also called text (discourse), focused on identifying the frequency with which certain data appeared for its subsequent synthesis and interpretation. According to the methodological structure of category-wise qualitative analysis, the data were deconstructed, and later gathered in a unit (analytical) structure that allowed identification of its elements (synthesis) [31].

The a priori categorisation (protocol of codification) was defined as follows:

Forms of institutionalisation (column 1): (1) Normative or advisory committees; (2) Committees of professional medical associations; (3) Committees of care and hospital ethics; and (4) Committees of ethics in research.

Forms of governance (column 2): ICB-Governance mechanisms and their elements have been recognised worldwide by the Universal Declaration of Bioethics (2005).

Institutional structure (column 3): These are the conditions that give legitimacy and consolidation to each ICB, as well as the form that in daily practice takes as a consultant or regulatory entity, whether in the decision of collective, substantive or political processes.

Political discourse (column 4): This content refers to the common object and a series of specific goals which are presented in the medical, scientific, technological, public, social and political fields.

Power mechanism (column 5): As a security device, it might be understood as the combination of knowledge-power-truth that reveals how legal, medical and political discourses can be translated into approved regulatory practices to exercise power not only over the bodies, but on the populations.

Symbolic role (column 6): This symbolic paper could be covered with a veiled power, or by a discursive power, a discourse capable of controlling some minds and in turn controlling some actions. At the beginning of the exploratory phase, the characterisation of some roles could represent the role of ICB as follows: Government elites, Power elite, Control mechanism, Intellectual and moral authority, Discussion forums and Passive actors [32].

Based on this model, it was intended to describe the process that would give way to the emergence of theoretical structures of this work; implicit in the material compiled and that would integrate it into a logical whole. A model capable of schematising the content of the information had already been constructed, essentially grouped into the following codes: categorisation, structuring, testing and theorising.

The final step of content analysis was the examination of the text/discourse of the ICB; they must be integrated into the relational framework to determine if they really act as government advisors that generate social value. Derived from the previous analysis, several details that gave consistency to the present exploratory work were revealed, for example, the role that public institutions have, as the expression of political forces through which societies propose to solve some of their collective problems. In this case, their role seems to be necessarily influenced by the rules and practices of the political system; however, in the vast majority of political systems, the democratic imperfection itself hinders the representation of institutions [33].

## Discussion

### What do we learn?: Insights on the case study

Institutional text/discourse has an important contribution in social reality. Its analysis was used to approach this reality through a linguistic process, which was used to see beyond the organisational practices. The central question of this work is: what is the role of the ICB in public health policies? To answer this question, it was necessary to dig deep into the text/discourse in order to produce and reproduce the response. In fact, other categories emerged as well as other socially constructed behaviours such as the legitimacy and resistance of institutional actors.

With this analysis, the relevance involved in the identification of texts and discourses revealed social constructions associated with pre-established texts and discourses that predetermine institutional policies and practices with the intention of strengthening its legitimacy [23].

On the other hand, the mapping of the a priory categories has been used for several purposes, such as the display of a complex structure, the communication of complicated ideas and the demonstration of connections between ideas [34]. The role that ICB have on health policies and which could modify the social, political and health dynamics, could be systematised using the Atlas.ti software. Once a code table was available, a map of preliminary categories could then generate a visualisation format using an alluvial diagram, seen in Fig. 5.

**Fig. 6:**
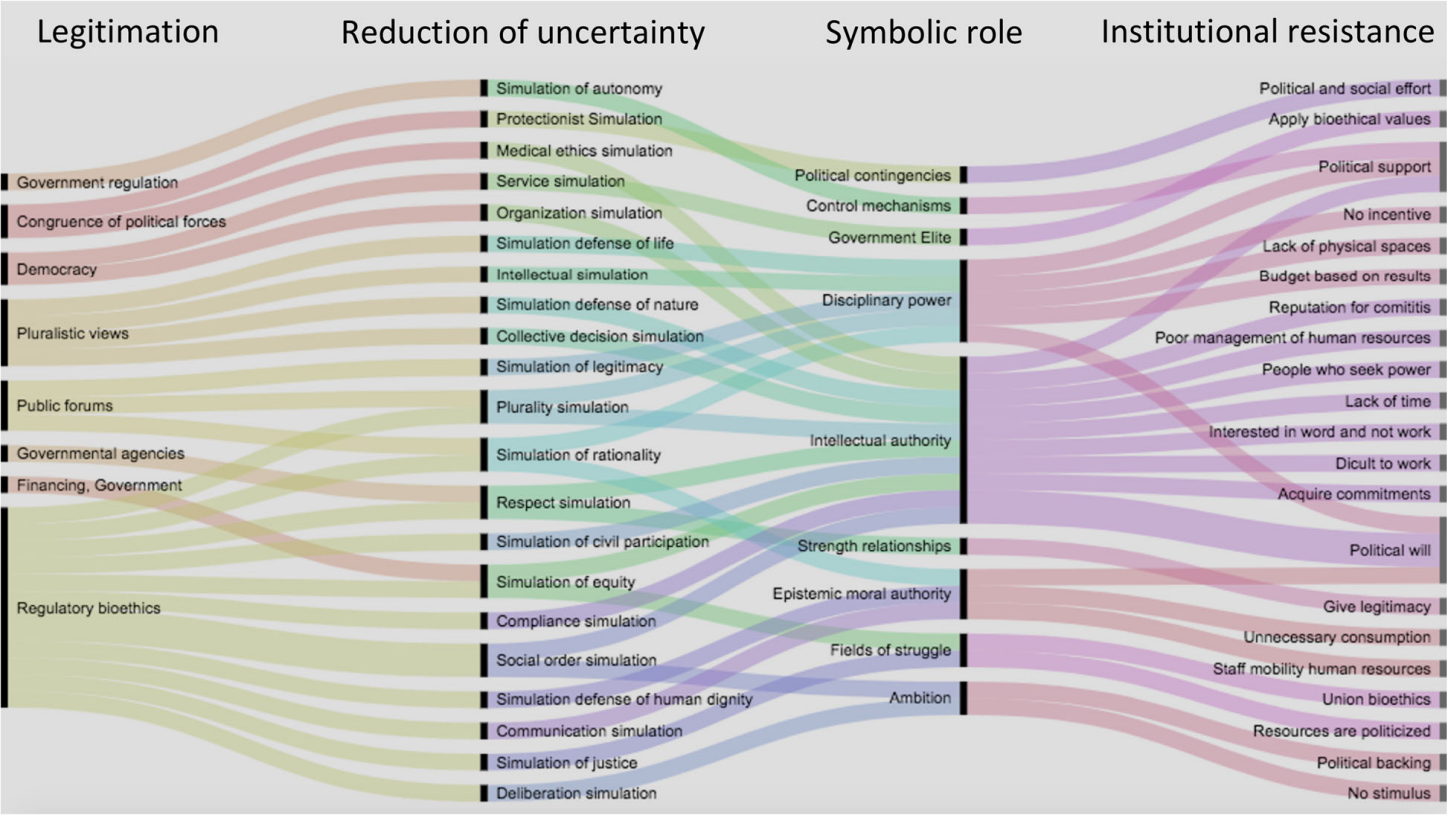
Alluvial diagram that shows the theoretical proposal and the emergent categories on the legitimisation and the institutional resistance: Columns: (1) Legitimisation, (2) Reduction of uncertainty, (3) Symbolic paper, (4) Resistance. In the column of codes of resistance, it can be seen that there are two that seem to be the most outstanding, namely political support and political will

When these a priory categories are applied to a critical text/discourse analysis, it may be necessary to recode the information and to re-run the analysis with the new codes and categories to redefine the research hypothesis and its interpretation. The result of this reorganisation forced the modification of the code table generated a priori, so that the category map was also remodelled and, at the same time, it was visualised by means of an alluvial diagram (RAW Graphs), as seen in Fig. 6.

Now, the main difference between Figs. 5 and 6, was the replacement of some columns with emerging categories in order to reduce uncertainty resulting in the disaggregation into various codes that we interpreted as being based on the concept of simulation, being this a result of the applied methodology. A question arose as to how can this theoretical concept could be seen or applied to reality. The domination of capitalism demanded a bioethical reflection based on a social analysis. Identifying the uncertainty in social, biological and political systems became essential to understand the central role of the institutions, both to overcome this uncertainty and to propose strategies of struggle.

The institutionalisation of bioethics has been described as a response to a mixture of demands for emerging public concerns (including those about advances in technology and also unethical practices) and to change political contexts in which questions about the mass data or the value of life was debated and translated into principles of rules to guide public life [35]. The formalism and the attachment to legality have been part of a political text/discourse, but not of the constant practice of the system [36]. Hence, it conforms to a sort of simulation.

Consequently, a question arose regarding the reconsideration of the case study exemplified in this article, as follows: Is progress being made in this regard or is it simply the appearance of giving attention to these uncertainties, by masking an irritating problem with a promising text/discourse, and at the same time confined to being the object of a generalised political-social control? This question had a close relationship with the case study questions: what is the role of the ICB in public health policies? How does the existing institutional arrangement work? What faculties, scope and limitations of power, as well as the exercise thereof, have been granted to these institutions? And what political and social tendency does it show when exercising its authority over matters that can directly affect health and life?

Based on this approach, it could be argued that institutions cannot directly affect policy outcomes, except by their impact on policy-making processes, from which they are designed, approved and implemented by stakeholders. Through the decision process, the institutions influence the adopted policies, in particular on the capacity to maintain inter-temporal commitments, the quality of the implementation, and the stability and credibility of the policies [37].

The challenge for this type of analysis to reveal this was then focused on linking citations of the text/discourse in larger conglomerates and higher level of abstraction while disaggregating the different dimensions or categories. This section of the discussion gave an account of how to connect the institutional theory with the forms of text/discourse analysis from a linguistic perspective, i.e. how to differentiate the discourse from the action of the political players. However, the question arose as to what would be the best way to prove that this would be true. One of the strategies employed was to speculate whether the role of ICB had some features of clientelism, patrimonialism, patronage, simulation or authoritarianism, rather than to only analyse the way in which the dynamics between the institution and government structure affect the results of public policies. Additionally, we intended to assess if the issues that generated uncertainty within the framework of bio-politics were priorities for the existing institutional arrangement or simply seemed to give attention to these in terms of maintaining social control. Again, a form of political simulation.

Hence, in order to verify the role of the ICB to reduce uncertainty, the concept of simulation was taken as an example to speculate on how these bodies could respond to the issues of concern, both to society and to national policy, based on the hypothesis that, if these are not attending to, then a social, scientific and even bio-political lack of control on the international scene may occur. The concept of institutional simulation could be used to characterise the authoritarian system, as well as its liberal democracy.

Therefore, derived from a deeper, more rigorous and critical reading of the information obtained, we made an integration and proposed that a column could be added to the theoretical map originally considered regarding the tentative form in which the ICB address the issues of uncertainty that could affect health and life. At the same time, it was proposed that some other columns were no longer necessary for this stage. Thus, the visualisation of the first map of categories (Fig. 5), was modified in this second stage as a result of textual and discursive citations that emerged inductively from the information collected Fig. 6. In the column of codes of resistance, it can be seen that there are two stances that seem to be the most outstanding, namely political support and political will.

Finally, by integrating all the information and the way in which it was reconstructed in the category maps, it was possible to identify, in the text/discourse of the representatives of the ICB that were intervened, a tendency to fight against a system of appearance or simulation.

Derived from this analysis, a map of categories that would contain some of the concepts proposed to prove the traits of resistance, as well as the possible connection with the other codes and categories that emerged in text/discourse analysis, was reconstructed.

### How do we learn?: Some advantages and limitations of the proposed approach

Finally, we want to briefly discuss some of the advantages and limitations of the methodology just outlined and exemplified in the study of the role of ICB in public health policy. Namely, the use of computational literature retrieval and classification, the introduction of ontologies –in this case based on PubMed’s MeSH classifier keywords– to build semantic networks and the use of hybrid manual/automated methods for the critical analysis of discourse.

As we have already mentioned, the use of contemporary data science techniques, such as computer-aided semi-automated literature retrieval and classification, provides helpful results since it implies the elimination (or better, the reduction) of sampling biases, resulting from the tendency of researchers to look for information mostly from their preferred sources, some of them ideologically skewed.

There is also the advantage of increased focus, coming from the use of the ontological classification of the concepts, such that conceptual gaps are diminished. For instance, there may be concepts that are closely related but differently enunciated or named in varying cultural circles. The use of ontologies such as the one created by PubMed’s MeSH terms somehow anneals these differences by creating a common language.

Another advantage of the MeSH ontology is, of course, the fact that it allows us to build semantic networks on a global, non-biased way, as terms are linked not by a personal opinion but from a kind of scholarly agreement arising from a large body of peer-reviewed work. Network analysis gives rise to emergent features coming from somehow unexpected conceptual connections that, as in the case of the simulation hypothesis on the role of ICB, are not evident from the study of single instances.

Combining these advantages with the use of hybrid approaches to the critical analysis of discourse allow for unbiased, but still individualised (i.e. human) critiques of the literature in a way that makes evident how the objective and subjective elements of discourse analysis are being carried out. This is highly desirable in the analysis of public policy, and particularly useful in decision-making scenarios.

After enumerating the advantages of using the present approach, we should however mention that, as it is clear, there is no study free of limitations. One particular limitation of this approach is indeed the use of pre-determined ontologies, namely the MeSH system of classifiers. MeSH terms constitute a detailed and structured ontology, which is useful for automated text classification, as it was designed with this in mind. In this regard, specific concepts, relevant to healthcare policy issues, may not be appropriately rendered to a unique MeSH term. As a consequence, the specificity in our description may be partially compromised.

The documental corpus belongs completely to literature published and indexed in the PubMed/Medline database. By this mere fact, there are a number of publication biases introduced. One particularly relevant bias is given by over-representation of papers from the top publishing countries on the subject. Many of them are actually developed countries with their characteristic issues in healthcare policy, which may not reflect the different facets of policy-making and implementation at a worldwide level.

## Conclusions

In this article, based on an exhaustive analysis of the literature, following the conceptual tenets of collective health, we developed a novel methodological approach to the problem of critical content analysis. This alternative, which combines novel methodologies of computational data and literature mining and semantic network analysis as well as hybrid manual/automated analysis of discourse, was proposed to study the role of the ICB, as well as some of its expressions in policies, as already discussed.

The challenge of analysing the role of committees in particular, as public bodies, is mainly due to the fact that they are highly dynamic entities. Although their actions can activate transcendental political processes for society, the vast majority of these are intangible and difficult to determine.

This novel approach has allowed us to identify ‘simulation’ as one possible rationale behind the formation of ICB, i.e. one of the reasons behind the creation of ICB may be giving the impression of attending an ethical necessity –to oversee and protect life, society and nature– with political purposes.

We can conclude then that in some cases ICB are formed to attend some bioethical issues to prevent disturbances of the social and institutional order, i.e. to preserve the status quo.

In this work, we introduce a novel, pragmatic approach for the progressive, systematic analysis and exploration of large information corpora. These tools are useful for the study of qualitative data to improve the performance of institutional assessment, and public policy redesign. Interactive strategies are also helpful to perform systematic revisions of the literature, for pattern generation and codification schemes, and for diagrammatic approaches to build models evidencing interactions among concepts and categories not defined a priori.

## Additional file

Additional file 1 **Interactive network files**. Interactive network files with all statistical and topological analyses. This is a Cytoscape.cys session. In order to open/view/modify this file please use the freely available Cytoscape software platform, available at http://www.cytoscape.org/download.php. (SIF 3413 kb)

## Abbreviations

CQAD: Computerized qualitative analysis of discourse
HCIPP: Health care institutions policies and practices
ICB: Institutional committees of bioethics
MEDLINE: Medical literature analysis and retrieval system online
MeSH: Medical subject headings
NSLR: Non-systematic literature reviews
QRM: Qualitative research methodology
